# Childhood maltreatment must lead to hate? The relation between childhood maltreatment and social mindfulness among college students: the roles of self-compassion, shyness and hostile attribution bias

**DOI:** 10.3389/fpsyg.2025.1447043

**Published:** 2025-03-17

**Authors:** XiaoYi Wang, GuangLan Yang, WeiJie Meng

**Affiliations:** ^1^College of Education, Ludong University, Yantai, China; ^2^Institute for Education and Treatment of Problematic Youth, Ludong University, Yantai, China; ^3^Shandong Business Institute, Yantai, China

**Keywords:** childhood maltreatment, social mindfulness, shyness, hostile attribution bias, self-compassion, moderated mediation effect

## Abstract

**Background:**

Childhood maltreatment represents a significant distal risk factor for the social adaptation and development of children and adolescents. However, the impact of childhood maltreatment on individuals’ social mindfulness—an emerging form of “effortless” prosocial behavior—remains largely unexplored.

**Objective:**

To address the gap in understanding the relationship between childhood maltreatment and social mindfulness, To address the gap in understanding the relationship between childhood maltreatment and social mindfulness, we conducted a cross-sectional study to clarify their association and explore potential influencing factors.

**Methods:**

In this study, 611 undergraduates were surveyed to complete a series of questionnaires including Childhood Maltreatment questionnaire-28 item Short Form(CTQ-SF), 17-item Social Mindfulness Self-report Scale(SMSRS), Shyness Scale, Chinese Version of Word Sentence Association Paradigm for Hostility Scale(CV-WSAP-Hostility), Chinese Version of Self-Compassion Scale(CV-SCS). And a moderated mediation model was constructed based on the data results.

**Results:**

Childhood maltreatment negatively predicts social mindfulness and exerts its influence through self-compassion as a mediator. Additionally, shyness and hostile attribution bias (HAB) were found to have significant independent and interactive moderating effects. Specifically, the negative impact of childhood maltreatment on self-compassion and social mindfulness diminished as levels of both shyness and HAB increased.

**Conclusion:**

This study demonstrates that the design of intervention programs for individuals with childhood maltreatment should fully consider the “dual-edged sword” effects of their shy personality traits and HAB and the potential for iatrogenic effects.

## Introduction

Social mindfulness refers to an interpersonal form of benevolence characterized by consideration and awareness of others’ needs in decision-making, with minimal or no cost to oneself (Van Doesum et al., 2013, 2021). It represents a low-cost prosocial behavior that prioritizes interpersonal kindness over material gains (Van Doesum et al., 2021). This mindset plays a key role in fostering interpersonal harmony and societal cohesion. At the micro level, it enhances interpersonal favorability and cooperation by recognizing and valuing others’ needs, while at the macro level, it supports the creation of democratic, respectful, and trust-based communities. Given its significant social implications, while existing research has primarily focused on exploring the mechanisms of social mindfulness in general populations, it is imperative for future investigations to extend their attention to vulnerable groups who may exhibit deficiencies in social mindfulness capacities. Given its broad social significance, most research has focused on social mindfulness within general populations, yet further investigation is needed on vulnerable groups that may lack these capacities. Research has shown that childhood maltreatment has lasting negative effects, extending from early childhood into adulthood, often leading to developmental challenges and maladaptive outcomes (Flynn et al., 2014; Greene et al., 2020). The college period represents a critical developmental stage characterized by significant challenges and risks, primarily stemming from students’ transition from family dependence to broader social engagement. The investigation of mechanisms underlying college students’ social mindfulness holds critical significance when examined through both individual developmental and societal functioning lenses.

### Childhood maltreatment and social mindfulness

Childhood maltreatment encompasses emotional abuse, physical abuse, neglect, and sexual abuse occurring before age 18 (Johnson et al., 1999). Empirical evidence demonstrates that such early adversity exerts profound and enduring effects on individuals’ self-concept development, emotional regulation, and behavioral patterns (Mell et al., 2021). Specifically, maltreatment impairs cognitive functioning, disrupts interpersonal relationships, and reduces prosocial behaviors, such as altruism and social trust. Conversely, social mindfulness—conceptualized as an altruistic signaling mechanism in interpersonal contexts (Dou et al., 2018; Van Doesum et al., 2013)—has been found to facilitate trust-building and cooperative social dynamics. A critical question is whether the effects of childhood maltreatment on social mindfulness mirror those observed in altruistic behaviors. While current research has yet to empirically support this link, emerging findings challenge the simplistic view that childhood adversity universally undermines prosocial capacities.

Three key considerations warrant attention: First, post-maltreatment growth, as outlined in the Altruism Born of Suffering (ABS) framework (Vollhardt, 2009), suggests that significant adversity can paradoxically promote prosocial development through enhanced socio-cognitive processes like empathy and perspective-taking (Vollhardt and Staub, 2011). Second, social mindfulness differs from traditional prosocial behavior in that it is a low-cost practice, requiring minimal behavioral investment as individuals become attuned to others’ needs (Yan et al., 2022). While traditional prosocial behaviors often involve substantial psychological costs (Song et al., 2018), social mindfulness remains independent of resource-sharing paradigms, making it a more feasible and scalable approach (Zhao et al., 2022). This unique blend of prosocial essence with behavioral feasibility positions social mindfulness as a promising intervention target. These insights suggest that maltreatment-informed social mindfulness interventions, grounded in developmental psychopathology, may offer greater ecological validity and implementation efficacy compared to traditional prosocial training, ultimately contributing to enhanced societal trust and community resilience.

### The mediation role of self-compassion

Serving as an adaptive emotion regulation mechanism, self-compassion buffers against the negative effects of adverse experiences (Peter and Gazelle, 2017). Unlike trait-like characteristics, self-compassion is malleable, with empirical evidence supporting its enhancement through targeted interventions and skill development (Neff, 2023; Bian, 2019; Hofmann et al., 2011). In the context of maltreatment recovery, self-compassion helps individuals (a) cognitively reframe painful experiences, (b) develop awareness of universal suffering, and (c) build meaningful connections based on shared humanity. This process fosters heightened empathy in maltreatment-exposed individuals, which in turn enhances altruistic behaviors and supports the development of social mindfulness through increased awareness of communal needs. Research further indicates that higher empathy in maltreated populations is associated with psychological resilience (Luo et al., 2021) and prosocial behaviors such as social support, trust-building, and emergency intervention (Welp and Brown, 2014). Given the well-documented therapeutic benefits of self-compassion for survivors of childhood maltreatment and its role as a relational catalyst, the current study examines its mediating role in the pathway from childhood maltreatment to social mindfulness, highlighting potential applications for maltreatment-informed social mindfulness enhancement programs.

### The moderation role of shyness and hostile attribution bias

Shyness, defined as a pervasive pattern of social inhibition and hypersensitivity to interpersonal evaluation (Henderson and Zimbardo, 2001), results from complex interactions between internalized psychological distress and impaired social functioning. According to the metacognitive model and the social fitness framework, shyness is characterized by maladaptive self-appraisals, excessive self-monitoring, and significant difficulties in social adjustment and prosocial behavior (Li and Li, 2023). Research indicates that the development of shyness is linked to poor parent–child relationships during early childhood (Doyle and Cicchetti, 2017), with childhood maltreatment exacerbating the emergence of extreme shyness, marked by heightened rumination and reactive aggression. Empirical studies also show a strong negative correlation between shyness and conventional prosocial behaviors (Li et al., 2018). However, paradoxically, heightened shyness correlates with increased online helping behaviors under conditions of reduced social evaluation pressure (Han et al., 2016). This apparent paradox suggests that shy individuals’ capacity for mindful prosocial expression may be selectively inhibited by context-dependent social appraisal mechanisms rather than reflecting global prosocial deficits.

Hostile attribution bias (HAB) refers to the tendency to interpret ambiguous situations as hostile or threatening (Huppert et al., 2007). Empirical evidence supports that the formation and development of hostile attribution bias have been found to be significantly associated with early harsh parental disciplinary practices (e.g., physical punishment, verbal criticism) and maladaptive attributional styles (Lee et al., 2020). Individuals exposed to harsh parenting may develop internal working models characterized by hostile perceptions of others, demonstrating a tendency to attribute negative intentions in ambiguous social contexts (Healy et al., 2015). Grounded in the Social Information Processing Model (Crick and Dodge, 1994), when individuals consistently interpret ambiguous social cues as hostile rather than benign, this cognitive pattern becomes a stable personality trait that influences behavioral responses (Dodge, 2006). Empirical evidence further demonstrates that attributional retraining programs, which promote prosocial interpretations, can enhance prosocial biases, leading to reduced anger and aggression, as well as improved well-being (Bockstaele et al., 2019). Therefore, the present study aims to explore the moderating roles of two stable individual difference factors—socio-emotional traits (operationalized as shyness) and cognitive processing tendencies (manifested as hostile attribution bias)—in shaping the relationship between early adverse experiences and subsequent social mindfulness capacities.

### The present study

**Figure 1:**
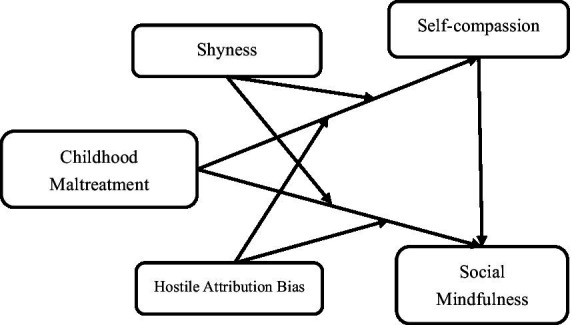
The proposed theoretical model.

The present study aims to explore the relationship between childhood maltreatment and social mindfulness among college students, mainly focusing on the mechanism of shyness, hostile attribution bias and self-compassion during this process. The proposed hypotheses are as follows: (H1) Childhood maltreatment experiences significantly influence social mindfulness among college students. (H2) Self-compassion mediates the relationship between childhood maltreatment and social mindfulness in college students. (H3) Shyness moderates the associations between childhood maltreatment and both self-compassion and social mindfulness in the proposed mediation model. (H4) Hostile attribution bias moderates the relationships between childhood maltreatment and both self-compassion and social mindfulness within the mediation framework. The hypothesis model is depicted in Figure 1.

## Method

### Participants

Based on the *a priori* sample size for structural equation models references (Soper, 2022), we applied G*power software to determine the sample size. Using an expected medium effect size (*f*^2^ = 0.15), an alpha level of 0.05, and a desired statistical power of 0.8., the analysis indicated that a total sample size of 85 participants would be required to detect the expected differences with adequate power. A total of 643 adolescents were recruited from a college in Shandong Province, China, using stratified random cluster sampling. Participants who had a response time of less than 4 min or failed to complete all items (*n* = 32) were excluded from the analysis. The final sample consisted of 611 participants (43.9% female), with ages ranging from 16 to 22 years. The participants’ living areas were as follows: 65.6% from rural areas, 22.3% from towns, and 12.1% from cities. Parental education levels indicated that 95.1% of fathers and 96.7% of mothers had a high school education or lower.

### Measures

#### Childhood maltreatment

Childhood maltreatment was assessed using the Chinese version of the Childhood Maltreatment Questionnaire Short Form (CTQ-SF) (Bernstein et al., 2003). The CTQ-SF consists of 28 items (seven of which are reverse-scored to assess response consistency) and measures experiences of childhood maltreatment across five dimensions: emotional abuse, emotional neglect, physical abuse, physical neglect, and sexual abuse. A sample item is: “Someone in my family said mean or insulting words to me before I was 16 years old.” Participants reflected on their experiences before the age of 16 and responded on a 5-point scale (1 = never, 5 = always). The total childhood maltreatment score is the sum of scores across all subscales, with higher scores indicating more frequent maltreatment. The Cronbach’s alpha for the full scale in the current study was 0.85, and for the five dimensions, it ranged from 0.65 to 0.76.

#### Social mindfulness

Social Mindfulness Self-report Scale (SMSRS) developed by Tian et al. (2021) was used to measure social mindfulness against the background of Chinese culture. The 17-item SMSRE contains two second-order factors (agreeableness and extraversion) and four first-order factors (kindness and respect, humility, optimism and open-mindedness, tolerance and understanding) for the internalization of social mindfulness. Participants respond on a 5-point score ranging from 1 = not at all true of me to 5 = very true of me (e.g., “I’ll not publicize it after I did something good.”). The sum of the four factors is the total score of social goodness. Cronbach’s alpha for the scale in the current study was 0.90, and Cronbach’s alpha for the four dimensions was 0.67 ~ 0.81.

#### Shyness

Shyness scale for Chinese junior high school students developed by Chen et al. (2015) was used to measure the level of shyness of the students. This scale consists of five dimensions: self-expression shyness, shyness toward stranger, shyness for negative social evaluation, shyness toward the opposite sex, humility shyness. Participants respond on a 5-point score ranging from 1 = not at all true of me to 5 = very true of me (e.g., “When I am noticed by the opposite sex, I feel blushed and embarrassed.”). Higher values represent that participants had higher level of shyness. Confirmatory factor analysis was performed because of the differences in the age of the participants. CFA model yielded acceptable fit indices (CFI = 0.92, RMSEA = 0.06, IFI = 0.93, NFI = 0.90). It indicates that this questionnaire is also suitable for the college students. Cronbach’s alpha for the scale in the current study was 0.94, and Cronbach’s alpha for the five dimensions was 0.60 ~ 0.89.

#### Self-compassion

The Chinese Version of Self-Compassion Scale (CV-SCS) revised was used to assess the level of the students’ self-compassion (Chen et al., 2011). This questionnaire comprises 26-item with six dimensions, namely self-kindness, self-judgment, common humanity, isolation, mindfulness and over-identification. Responses are given on a 5-point scale from (1 = almost never, 5 = almost always). Items representing uncompassionate responses to suffering are reverse-coded. Then means are calculated for each subscale, and grand mean is calculated that represents an overall measure of self-compassion. Higher values represent that participants had higher level of self-compassion. Cronbach’s alpha for the scale in the current study was 0.87, and Cronbach’s alpha for the six dimensions was 0.65 ~ 0.80.

#### Hostile attribution bias

The Chinese version of Word Sentence Association Paradigm for Hostility Scale (WSAP-Hostility) revised by Quan (2019) will be adopted to measure hostile attribution bias. In current study, a total of 16 items with a 6-point scale from (1 = not relevant at all, 6 = very relevant) are used to evaluate the correlation between hostile words and sentences in ambiguous situations, and the mean of the final score was the score of hostile attribution bias. Cronbach’s alpha for the scale in the current study was 0.93.

#### Analyses strategy

SPSS 26.0 was used to calculate descriptive statistics and correlations. We then use confirmatory factor analysis (CFA) in AMOS 27.0 to check the factorial validity of the scales. The SPSS macro program compiled by Hayes (2013) was used to test the moderated mediation analysis.

## Results

### Common methodological deviation test

Harman single factor method was used to test the possible common methodological deviation (Podsakoff et al., 2003). A total of 25 factors were found to have eigenvalues greater than 1, among which the variance explained by the first factor was 17.55%, less than 40%. Therefore, there is no significant common methodological bias in current study.

### Variable correlations

This study included a sample of 611 college students. Table 1 shows the standard deviations and correlation coefficients for all study variables. Correlation analyses revealed three significant positive associations: between childhood maltreatment and shyness, childhood maltreatment and hostile attribution bias, and between shyness and hostile attribution bias. Additionally, self-compassion was positively correlated with social mindfulness. Both self-compassion and social mindfulness exhibited significant negative correlations with childhood maltreatment, shyness, and hostile attribution bias.

**Table 1:** Descriptive statistics and bivariate correlations for all study variables (*n* = 611).

	*M*	*SD*	1	2	3	4	5
1 Childhood maltreatment	34.95	9.26					
2 Shyness	86.23	21.82	0.23^**^				
3 Hostile attribution bias	41.39	16.72	0.18^**^	0.24^**^			
4 Self-compassion	87.87	12.89	−0.37^**^	−0.52^**^	−0.36^**^		
5 Social mindfulness	65.85	9.52	−0.33^**^	−0.30^**^	−0.15^**^	0.48^**^	

^*^p < 0.05, ^**^p < 0.01, ^***^p < 0.001.

### The moderated mediation effect

**Figure 2:**
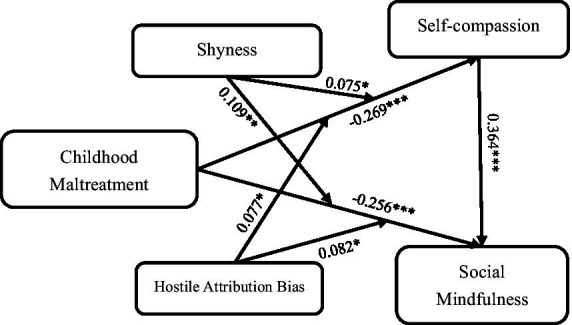
The moderated mediation effect model and parameter standardization estimate. *p < 0.05, **p < 0.01, ***p < 0.001.

The results of correlation analysis meet the statistical requirement for testing mediation effects (Wen and Ye, 2014). The SPSS macro program^1^ compiled by Hayes (2013) was used to test the moderated mediation analysis with model 10. The regression analysis results (see Table 2 and Figure 2) revealed that childhood maltreatment significantly and negatively predicted social mindfulness (*c’* = −0.256, *p* < 0.001) and self-compassion (*a* = −0.269, *p* < 0.001). In contrast, self-compassion significantly and positively predicted social mindfulness (*b* = 0.364, *p* < 0.001). The mediation analysis indicated that the indirect effect of self-compassion on the relationship between childhood maltreatment and social mindfulness, *ab* = −0.098, with a 95% confidence interval of [0.277, 0.185]. The proportion of the relative mediation effect was *ab*/(*ab* + *c’*) = 27.67%.

**Table 2:** Results of the moderated mediation analysis.

Predictors	Equation 1 (DV: self-compassion)			Equation 2 (DV: social mindfulness)		
	*SE*	*β*	*t*	*SE*	*β*	*t*
Childhood maltreatment (CM)	0.035	−0.269***	−7.681	0.397	−0.256***	−6.464
Shyness (SH)	0.034	−0.413***	−12.307	0.041	−0.075	−1.844
Hostile attribution bias (HAB)	0.034	−0.206***	−6.146	0.037	0.034	0.902
Childhood maltreatment × SH	0.031	0.075*	2.395	0.034	0.109**	3.200
Childhood maltreatment × HAB	0.032	0.077*	2.444	0.034	0.082*	2.371
Self-compassion (SC)				0.044	0.364***	8.248
*R* ^2^		0.398			0.300	
*F*		39.831***			23.327***	

DV, dependent variable. Standard errors are reported in parentheses. ***p < 0.001, **p < 0.01, *p < 0.05.

Furthermore, shyness and hostile attribution bias were included as moderators in the regression analysis. The results demonstrated that the interaction term between childhood maltreatment and shyness significantly predicted self-compassion (*β* = 0.075, *p* < 0.05) and social mindfulness (*β* = 0.109, *p* < 0.001). Similarly, the interaction term between childhood maltreatment and hostile attribution bias also significantly predicted self-compassion (*β* = 0.077, *p* < 0.05) and social mindfulness (*β* = 0.082, *p* < 0.05). The findings demonstrate that shyness and hostile attribution bias, as stable personality traits and cognitive patterns, play a moderating role in the associations linking childhood maltreatment to both social mindfulness and self-compassion.

**Figure 3:**
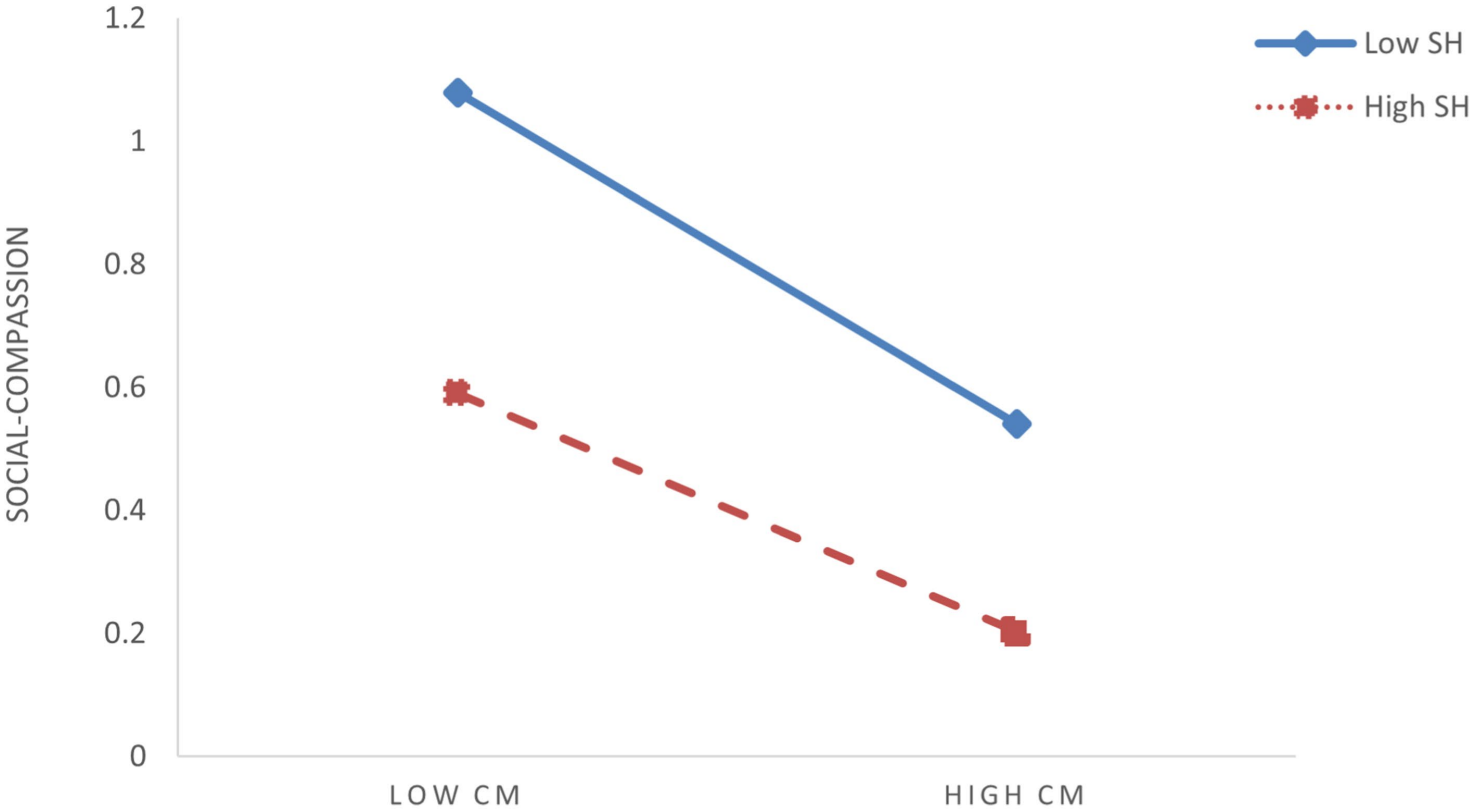
The moderating effect of shyness on the relationship between childhood maltreatment (CM) and social-compassion (SC).

**Figure 4:**
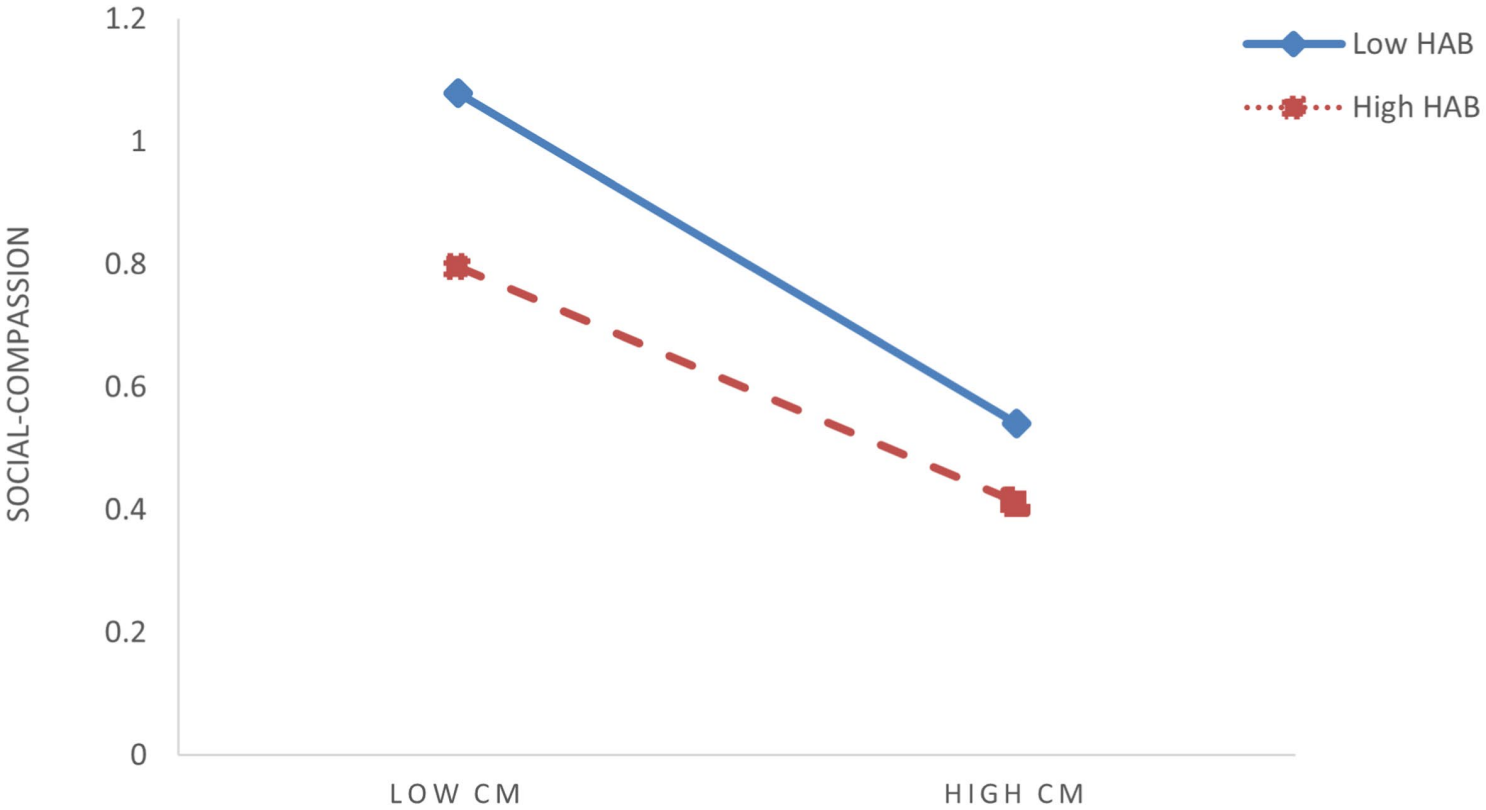
The moderating effect of HAB on the relationship between childhood maltreatment (CM) and social-compassion (SC).

**Figure 5:**
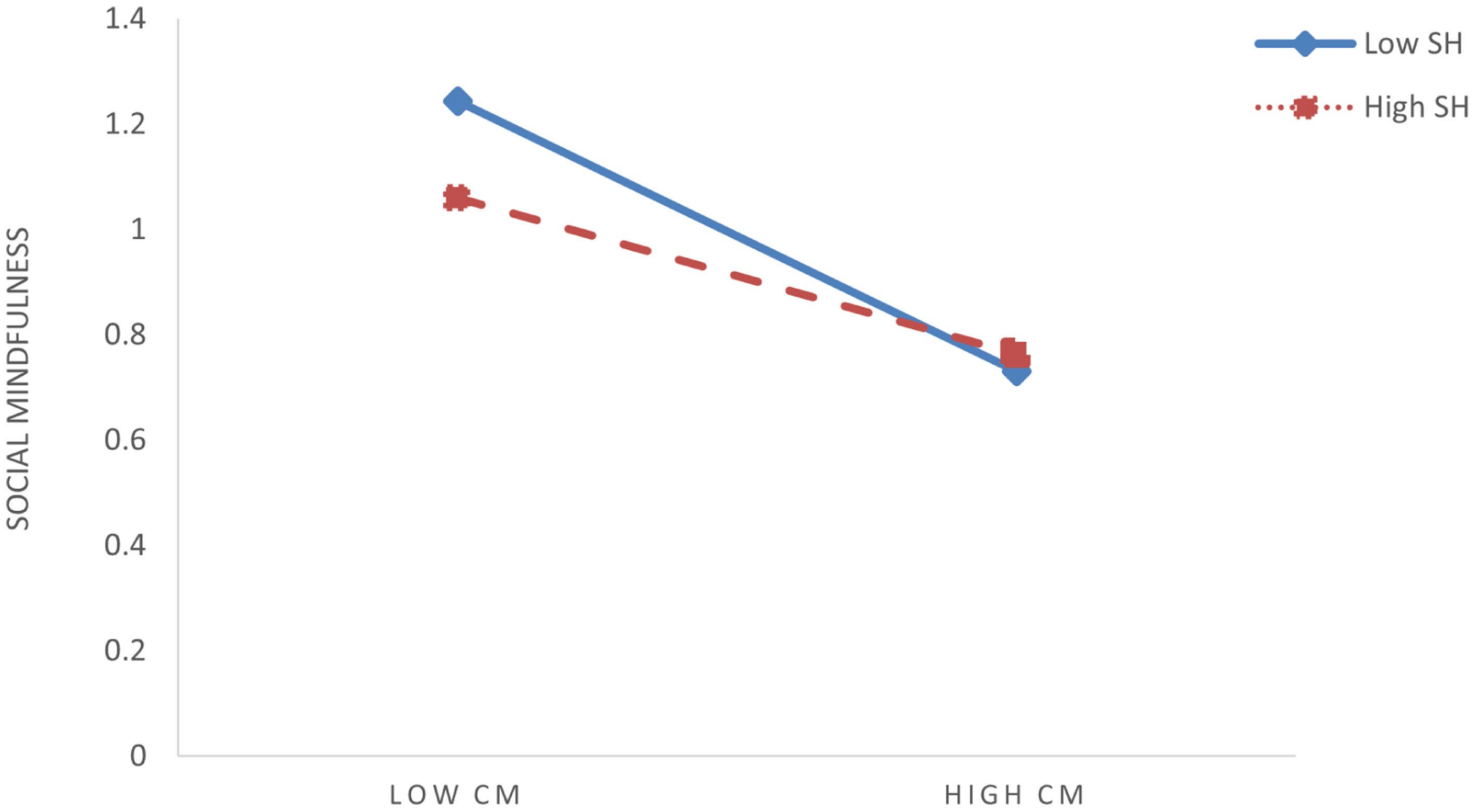
The moderating effect of shyness (SH) on the relationship between childhood maltreatment (CM) and social mindfulness (SM).

**Figure 6:**
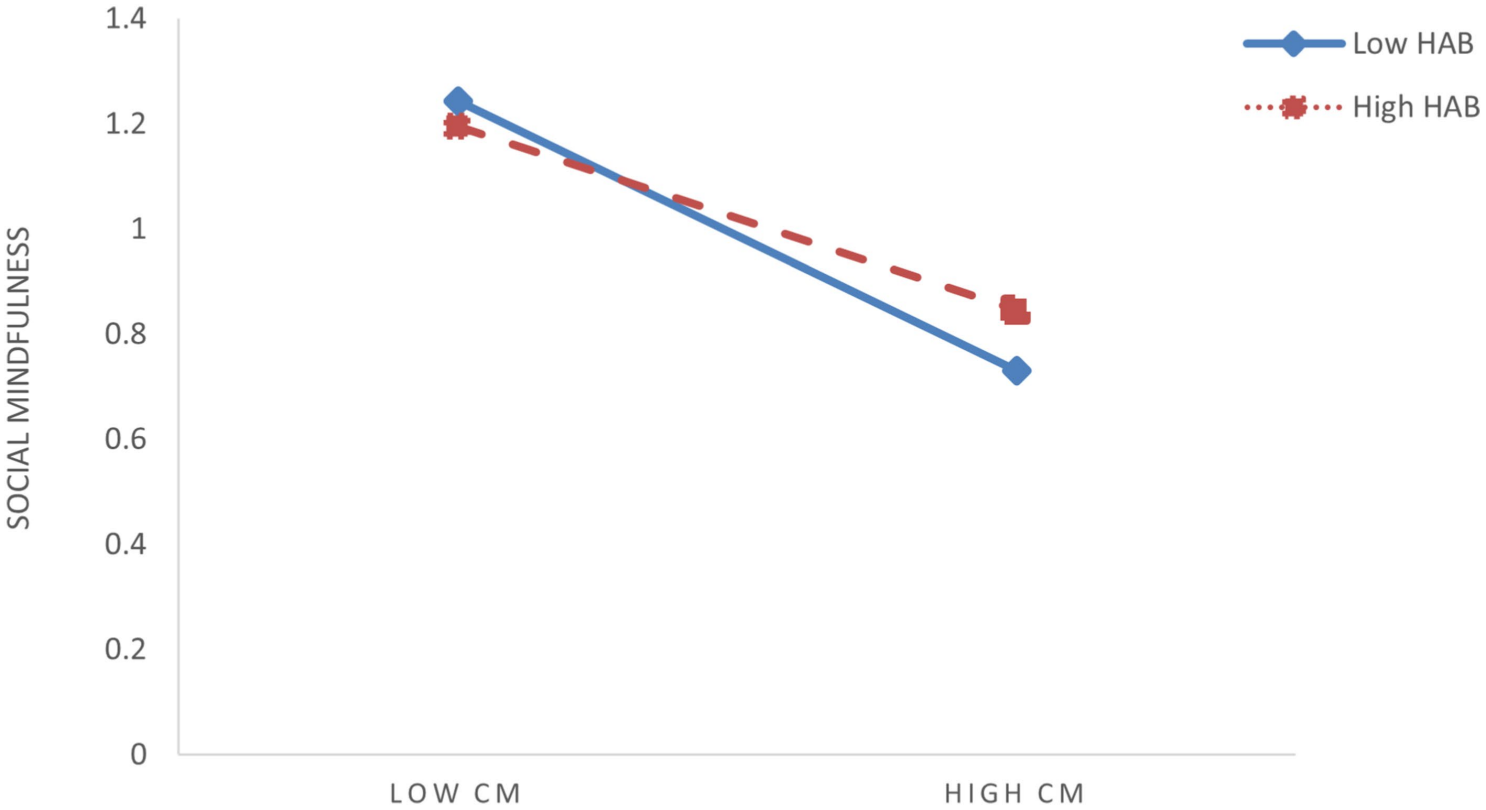
The moderating effect of HAB on the relationship between childhood maltreatment (CM) and social mindfulness (SM).

To further elucidate the moderating effects of shyness and hostile attribution bias, we conducted simple slope analyses by categorizing participants into high (M + 1SD) and low (M – 1SD) groups based on ±1 standard deviation from the mean scores of these two moderators. To visualize interaction patterns, simple effect analyses were conducted (Figures 3–6). The analysis revealed differential patterns based on shyness levels: For individuals with low shyness, childhood maltreatment significantly negatively predicted self-compassion (*β*_simple_ = −0.344, *t* = −6.585, *p* < 0.001). Conversely, this negative predictive effect was attenuated in highly shy individuals while remaining significant (*β*_simple_ = −0.194, *t* = −4.737, *p* < 0.001), indicating that increased shyness levels weakened the detrimental impact of childhood maltreatment on self-compassion (Figure 3). Similarly, childhood maltreatment showed significant negative effects on self-compassion under low HAB conditions (*β*_simple_ = −0.347, *t* = −6.884, *p* < 0.001). Although this negative association persisted at high HAB levels, its magnitude was substantially reduced (*β*_simple_ = −0.192, *t* = −4.367, *p* < 0.001), demonstrating HAB’s buffering role against the adverse effects of childhood maltreatment (Figure 4). Furthermore, the pattern extended to social mindfulness outcomes. Childhood maltreatment exerted strong negative effects on social mindfulness at low shyness levels (*β*_simple_ = −0.365, *t* = −6.246, *p* < 0.001). This relationship, while remaining statistically significant, showed progressive attenuation with increasing shyness (*β*_simple_ = −0.148, *t* = −3.276, *p* < 0.001) (Figure 5). Notably, the moderating role of HAB mirrored the patterns observed with shyness. Higher HAB levels progressively diminished childhood maltreatment’s negative impact on social mindfulness, with significant effects persisting at both low (*β*_simple_ = −0.338, *t* = −5.983, *p* < 0.001) and high HAB conditions (*β*_simple_ = −0.175, *t* = −3.629, *p* < 0.001) (Figure 6), a significant synergistic interaction emerged between shyness and HAB (Δ*R*^2^ = 0.024, *p* < 0.001). The combined moderating effect revealed maximum negative impact of childhood maltreatment on social mindfulness when both shyness and HAB were low (*β*_simple_ = −0.447, *p* < 0.001), contrasting with minimal and non-significant effects when both moderators were elevated (*β*_simple_ = −0.066, *p* = 0.162).

## Discussion

### Childhood maltreatment and social mindfulness among college students

The results of this study indicate that childhood maltreatment significantly and negatively predicts social mindfulness. Specifically, individuals exposed to higher levels of childhood maltreatment exhibit lower levels of prosocial orientation, supporting Hypothesis 1. These findings are consistent with prior research on the link between childhood maltreatment and prosocial behavior (Zhuo, 2022; Zhang, 2023; Xu and Yang, 2024) and highlight the lasting detrimental effects of childhood trauma as a distal risk factor for social adaptation and developmental trajectories (Wang et al., 2022; Zheng et al., 2023; Sun et al., 2024; Huang et al., 2024). Victims of childhood maltreatment are particularly vulnerable to a range of adverse psychosocial outcomes throughout both childhood and adulthood (Font and Berger, 2015). Empirical evidence indicates that college students from rural backgrounds report significantly higher levels of childhood emotional maltreatment, a pattern strongly linked to their socioeconomic status and familial cultural contexts (Hou et al., 2017; Huang et al., 2024). In the current study, participants reported higher rates of childhood physical and emotional neglect, which may be linked to their families’ socioeconomic conditions. Specifically, many participants’ parents are migrant workers with limited educational attainment, often preoccupied with securing livelihoods, leading to minimal parent–child interaction and reduced familial engagement. Furthermore, the relationship between childhood maltreatment and social mindfulness in this study mirrors its association with prosocial behavior (Zhang, 2023; Xu and Yang, 2024), suggesting that childhood maltreatment broadly impairs both helping thoughts and behaviors. However, although this study did not directly compare social mindfulness and prosocial behavior within the same research framework, insights from Van Doesum et al.’s (2013, 2021) work suggest that social mindfulness focuses more on subtle, context-specific decision-making, whereas prosocial behavior encompasses a broader range of actions. Social mindfulness may be more susceptible to the indirect effects of childhood trauma, while prosocial behavior could operate through different mechanisms. This limitation presents an opportunity for future research.

### The mediation effect of self-compassion

This study demonstrates that self-compassion mediates the relationship between childhood maltreatment and social mindfulness. Specifically, individuals who have experienced childhood maltreatment can mitigate its negative effects on social mindfulness by fostering their self-compassion abilities. This finding supports Hypothesis 2 and aligns with previous research suggesting that self-compassion acts as a protective factor against various emotional disorders (Peter and Gazelle, 2017; Wu et al., 2021). From the perspective of individual motivation, the desire to cultivate self-compassion is linked to pain-related stimuli, as individuals are inherently motivated to alleviate suffering (Gilbert, 2019). Just as childhood maltreatment has numerous proximal and distal adverse effects on individual development, the psychological and physical pain it causes may activate self-compassion, positioning it as a key factor in fostering altruism after suffering. From a socio-psychological standpoint, self-compassion also serves as a motivation for reciprocal exchange (Gilbert, 2019), helping individuals strengthen their connections with others, gain broader perspectives, and develop the capacity to care for others (Neff, 2023). Thus, self-compassion training is essential for individuals who have experienced childhood maltreatment. Such training can help reduce excessive self-criticism, build resilience, and empower individuals to face challenges, ultimately contributing to social harmony and stability.

### The moderating effects of shyness and hostile attribution bias

This study examines the impact of childhood maltreatment on self-compassion and social mindfulness, as well as the moderating roles of shyness and hostile attribution bias (HAB) in this relationship. The results show that childhood maltreatment significantly negatively predicts both self-compassion and social mindfulness, with self-compassion serving as a mediator. Additionally, shyness and HAB significantly moderate the effects of childhood maltreatment on self-compassion and social mindfulness, exhibiting a synergistic moderating effect. Hypotheses 3 and 4 were supported.

### Moderating role of shyness

The study shows that shyness, as an individual trait, can buffer the negative impact of childhood maltreatment on both self-compassion and social mindfulness, demonstrating a “double-edged sword” effect. In more positive contexts (i.e., low levels of childhood trauma), individuals with lower levels of shyness are more likely to exhibit higher social mindfulness and can enhance it through self-compassion. However, in more negative contexts (i.e., high levels of childhood trauma), high shyness acts as a protective factor, reducing the negative impact of childhood maltreatment on both self-compassion and social mindfulness. The Social Fitness Model of Shyness posits that shy individuals demonstrate self-enhancement bias in social contexts, characterized by overestimation of negative evaluations from others and underestimation of their own social performance, thereby contributing to maladaptive emotional responses, cognitive patterns, and behavioral tendencies (Henderson, 2014). This theoretical framework extends to explain the manifestation of social mindfulness in shy populations. Individuals with lower shyness levels, possessing better social adaptation capabilities, tend to exhibit higher levels of social mindfulness. However, the expression of these behaviors requires a supportive environment. In the context of childhood maltreatment, individuals with lower levels of shyness may become more vulnerable to external pressures, lacking introspection, which negatively impacts their self-compassion and social mindfulness. In contrast, highly shy individuals may internalize their pain, reducing sensitivity to external harm, which mitigates the negative effects of childhood maltreatment on both self-compassion and social mindfulness.

### Moderating role of hostile attribution bias

The analysis further reveals that hostile attribution bias (HAB) moderates both the initial stage of the mediation pathway and the direct effect pathway, demonstrating regulatory mechanisms analogous to those observed in shyness. These findings highlight the dual role of HAB in influencing social mindfulness among individuals with a history of childhood maltreatment. Specifically, under benign environmental conditions (i.e., low activation of childhood maltreatment), lower levels of HAB helped mitigate the negative effects of maltreatment on self-compassion and social mindfulness, whereas higher HAB levels hindered these adaptive outcomes. Paradoxically, under adverse conditions (i.e., high activation of childhood maltreatment), individuals with lower HAB exhibited diminished social mindfulness, while those with higher HAB demonstrated a protective resilience, attenuating the negative impacts of childhood trauma on both self-compassion and social mindfulness. This paradox may stem from differing cognitive appraisal patterns. High-HAB individuals tend to “rationalize” traumatic events, such as attributing blame to others, which reduces self-blame and fosters tolerance toward others. Consistent with Social Information Processing (SIP) theory (Dodge, 2006), HAB, as a maladaptive attributional style, may serve defensive functions in threat-laden contexts. However, it is important to note that this buffering effect is not entirely offset. The reason lies in the fact that although HAB may reduce emotional pain in the short term, chronic high HAB can lead to social isolation, interpersonal conflict, or mental health issues (e.g., depression), ultimately undermining its protective function.

### Joint moderating effects of shyness and hostile attribution bias

The findings of this study indicate that shyness and hostile attribution bias (HAB) jointly moderate the relationship between childhood maltreatment and self-compassion as well as social mindfulness. When both factors interact, the negative impact of childhood maltreatment on self-compassion and social mindfulness is further attenuated. The cognitive-behavioral model of social anxiety posits that individuals with social anxiety exhibit inherent negative cognitive patterns, characterized by maladaptive beliefs about themselves and their social environments. Empirical evidence supports that interventions targeting negative interpretation biases can significantly improve social adaptation in shy individuals (Tang, 2020), and there may be a shared emotional mechanism between shyness and HAB (Weeks et al., 2016). Fabes and Eisenberg (1992) proposed that higher emotional sensitivity and lower emotional regulation may contribute to the development of shyness, while hostile attribution bias is consistently associated with high emotionality and low emotional regulation (Smeijers et al., 2019). Thus, the synergistic moderating effect observed in this study may stem from the complementary cognitive and affective mechanisms of shyness and HAB. Specifically, shyness may reduce sensitivity to external stimuli, while HAB may alter cognitive appraisals of threatening events, together buffering the negative impacts of childhood maltreatment.

### Implications and limitations

This study investigates how childhood maltreatment, as a distal developmental risk factor, influences individuals’ social mindfulness capacities. The results reveal that self-compassion serves as a key mediating mechanism, suggesting that self-compassion training could be an effective intervention to help individuals with adverse childhood experiences manage psychological distress, foster post-traumatic growth, and improve prosocial functioning. Additionally, we systematically investigated the moderating roles of two stable individual difference factors—shyness (a socio-emotional dispositional characteristic) and hostile attribution bias (HAB, a cognitive processing tendency)—in shaping the relationship between early adversity and social mindfulness. The results demonstrated significant independent and interactive moderating effects of shyness and HAB. Specifically, the detrimental impacts of childhood maltreatment on self-compassion and social mindfulness attenuated progressively with increasing levels of both shyness and HAB. However, this buffering effect manifested a dual-edged nature, implying that while these traits may provide temporary psychological protection, they could also limit long-term socioemotional development. These results highlight the importance of considering boundary conditions and potential iatrogenic effects when designing trauma-informed interventions.

Nevertheless, this study has several limitations that require further attention. First, the cross-sectional design prevents causal inferences or the examination of the dynamic relationships between these variables over time. Additionally, the sample primarily consists of college students, which may limit the generalizability of the findings. Future research could explore how social mindfulness varies across individuals with diverse backgrounds, personality traits, and behavioral tendencies. Second, the reliance on self-report measures may introduce social desirability bias. Future studies could incorporate peer evaluations of social mindfulness or experimental designs that manipulate childhood maltreatment levels to improve the validity of the results. Third, while this study focused on social mindfulness, it did not account for prosocial behavior, which could serve as a relevant control variable in future research. Moreover, the selection of control variables, such as personality traits (e.g., extraversion and agreeableness), mental health factors (e.g., depression and anxiety), and cultural considerations, should be more thoroughly addressed in future studies to provide a more comprehensive understanding of these dynamics.

## Conclusion

This study draws the following conclusions:

1. Childhood maltreatment experiences have a significant impact on social mindfulness in college students.
2. Self-compassion mediates the relationship between childhood maltreatment and social mindfulness in college students.
3. Shyness and hostile attribution bias moderate the associations between childhood maltreatment, self-compassion, and social mindfulness within the proposed mediation model, with both factors exerting a joint moderating effect on the relationship between childhood maltreatment and both self-compassion and social mindfulness.

## Data Availability

The raw data supporting the conclusions of this article will be made available by the authors, without undue reservation.

## Footnotes

^1^ http://www.Afhayes.com
